# GW8510 Increases Insulin Expression in Pancreatic Alpha Cells through Activation of p53 Transcriptional Activity

**DOI:** 10.1371/journal.pone.0028808

**Published:** 2012-01-05

**Authors:** Dina Fomina-Yadlin, Stefan Kubicek, Amedeo Vetere, Kaihui Hu He, Stuart L. Schreiber, Bridget K. Wagner

**Affiliations:** 1 Chemical Biology Program, the Broad Institute of Harvard and MIT, Cambridge, Massachusetts, United States of America; 2 Department of Molecular and Cellular Biology, Harvard University, Cambridge, Massachusetts, United States of America; 3 Research Centre for Molecular Medicine of the Austrian Academy of Sciences, Vienna, Austria; 4 Department of Chemistry and Chemical Biology, Harvard University, Cambridge, Massachusetts, United States of America; 5 Howard Hughes Medical Institute, the Broad Institute of Harvard and MIT, Cambridge, Massachusetts, United States of America; University of Bremen, Germany

## Abstract

**Background:**

Expression of insulin in terminally differentiated non-beta cell types in the pancreas could be important to treating type-1 diabetes. Previous findings led us to hypothesize involvement of kinase inhibition in induction of insulin expression in pancreatic alpha cells.

**Methodology/Principal Findings:**

Alpha (αTC1.6) cells and human islets were treated with GW8510 and other small-molecule inhibitors for up to 5 days. Alpha cells were assessed for gene- and protein-expression levels, cell-cycle status, promoter occupancy status by chromatin immunoprecipitation (ChIP), and p53-dependent transcriptional activity. GW8510, a putative CDK2 inhibitor, up-regulated insulin expression in mouse alpha cells and enhanced insulin secretion in dissociated human islets. Gene-expression profiling and gene-set enrichment analysis of GW8510-treated alpha cells suggested up-regulation of the p53 pathway. Accordingly, the compound increased p53 transcriptional activity and expression levels of p53 transcriptional targets. A predicted p53 response element in the promoter region of the mouse *Ins2* gene was verified by chromatin immunoprecipitation (ChIP). Further, inhibition of Jun N-terminal kinase (JNK) and p38 kinase activities suppressed insulin induction by GW8510.

**Conclusions/Significance:**

The induction of *Ins2* by GW8510 occurred through p53 in a JNK- and p38-dependent manner. These results implicate p53 activity in modulation of *Ins2* expression levels in pancreatic alpha cells, and point to a potential approach toward using small molecules to generate insulin in an alternative cell type.

## Introduction

Autoimmune attack on pancreatic beta cells in type-1 diabetes results in insulin deficiency and an inability to maintain glucose homeostasis [1]. Inducing the production of insulin in other cell types has the potential to assuage diabetes pathogenesis. Pancreatic alpha cells are attractive candidates because of their secretory nature, their developmental proximity to beta cells, and their location within the islet of Langerhans [2]. Further, conversion of alpha cells to functional beta cells has already been demonstrated in mice by ectopic expression of a single transcription factor, PAX4, in the developing pancreas [3]. Therefore, we hypothesized that small molecule-mediated stimulation of insulin expression in alpha cells is a necessary initial step for insulin production that does not require viral delivery [4] of master-regulatory transcription factors, and could lead to an alternative therapeutic strategy for type-1 diabetes.

Insulin expression is largely restricted to pancreatic beta cells, but there are low levels of expression in extra-pancreatic tissues, such as the brain [5], [6] and the thymus [7]. Temporal and tissue-specific regulation of the insulin gene demonstrates complexity across species [8]. The *cis*-regulatory 400-base pair region flanking the transcriptional start site (TSS) is highly regulated, controlled by both beta cell-specific transcriptional regulators and general transcription factors with widespread tissue distribution [8]. To date, most pancreatic endocrine cell research has focused on rodent cell lines [9]. In contrast to humans, rodents have a two-gene insulin system, with *Ins2* similar to the human insulin gene, and *Ins1* the result of a duplication-transposition of a partially processed *Ins2* mRNA product that lost the second intron [10], [11]. Here, we focused on modulation of *Ins2* transcription in mouse alpha cells.

Previously, we reported a high-content screen to identify small-molecule inducers of insulin expression in alpha cells, and the discovery of a putative kinase inhibitor [12]. A more focused exploration of the effects of other kinase inhibitors on insulin expression in alpha cells led us to discover that GW8510, a compound annotated as a CDK2 inhibitor [13], also up-regulates *Ins2* expression. Through further characterization of GW8510's effects on alpha cells, we demonstrate involvement of the p53 signal transduction pathway in the modulation of *Ins2* expression levels. P53 has transcription-factor activity and performs most of its biological functions through direct regulation of downstream transcriptional targets [14]. It functions by binding to specific DNA sequences containing p53 response elements, which results in either activation or repression of promoter activity of target genes [15], [16]. Integration of upstream signals leads to a variety of cellular responses to p53 activation, ranging from cell-cycle arrest, to differentiation, to apoptosis [17], [18]. Because of its functional diversity and its importance in cell-fate decisions, p53 levels and activity are tightly regulated through positive and negative feedback [19]. The best characterized negative feedback loop involves an E3 ubiquitin ligase, MDM2 [20], which is up-regulated by increased p53 levels or transcriptional activity [21]. Many signal-transduction pathways converge on p53, but result in differential regulation of downstream targets [22]. Thus, we sought to examine the mechanism by which GW8510 activates the p53 pathway and up-regulates *Ins2* expression. We determined that p53 binds to the *Ins2* promoter in alpha cells, and that GW8510 increases *Ins2* expression by up-regulating p53 transcriptional activity in a JNK- and p38-dependent manner. These results suggest that modulating these pathways with small molecules could be part of a feasible strategy for generating insulin in an alternative cell type.

## Results

**Figure 1:**
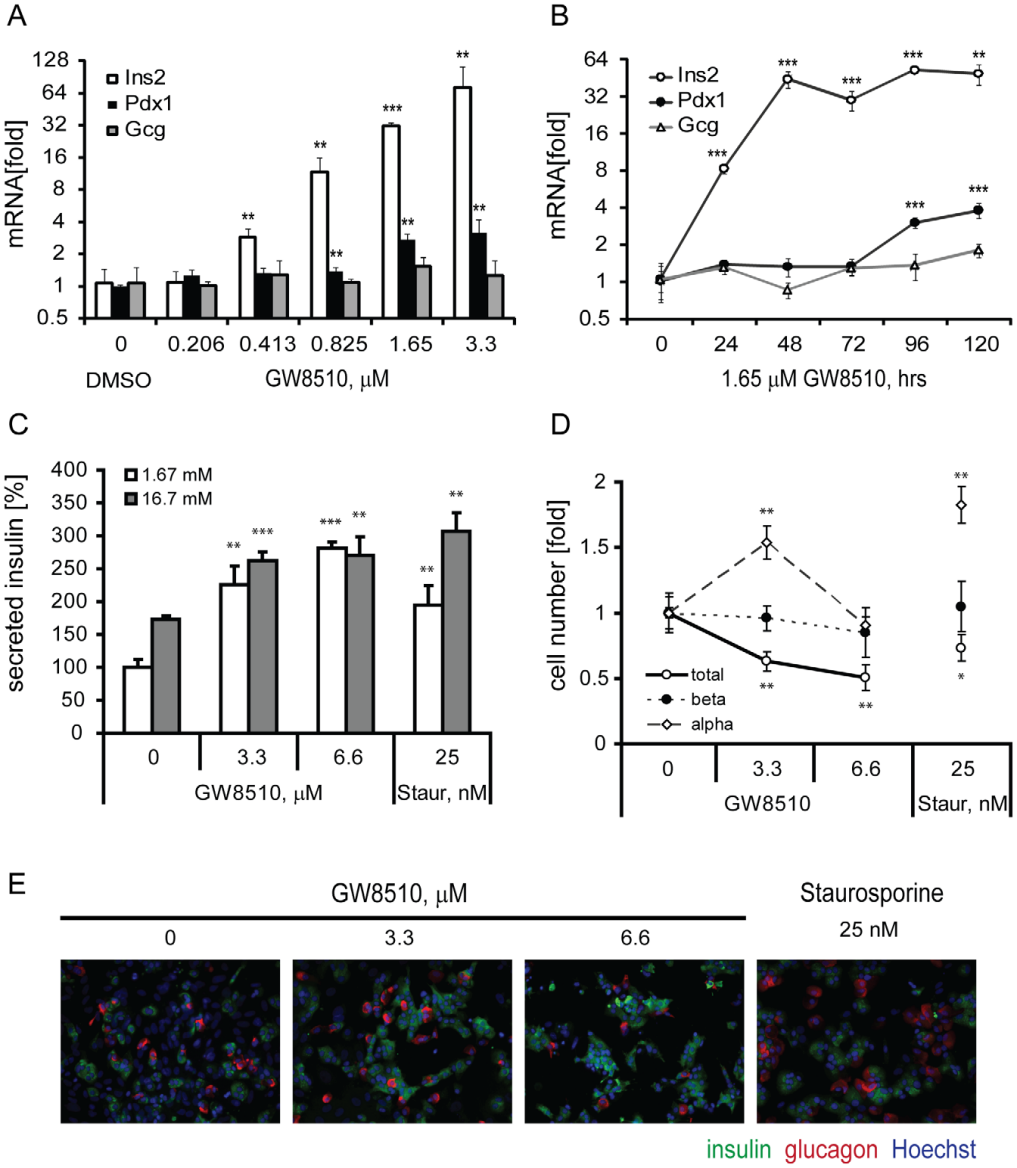
Effects of GW8510 treatment on mouse alpha cells and dissociated human islet cells. Pancreatic gene expression was measured by quantitative real-time RT-PCR (qPCR) following a (A) 5-day dose-response and (B) time-course with 1.65 µM GW8510. (C) Insulin secretion measurements in dissociated human islets following 5-day compound treatment at indicated concentrations. (D) Immunofluorescence analysis quantification of total cell numbers, measured by nuclear count, and numbers of alpha and beta cells, measured by glucagon and insulin staining, respectively, following compound treatment. (E) Representative images shown. All data represent the mean±SD of at least three experiments; *p<0.05, **p<0.01 and ***p<0.001.

Using high-content screening, we previously identified a compound, BRD7389, which induced insulin expression in alpha cells, and inhibited a wide variety of kinases biochemically [12]. To determine whether selective kinase inhibition could also induce insulin expression, we treated the mouse pancreatic alpha cell line, αTC1 clone 6 (αTC1.6), with the putative CDK2 inhibitor GW8510 for five days, and observed a dose-dependent induction of *Ins2* expression following treatment (Figure 1A). We observed similar effects when we used αTC1 clone 9 cells, which express more insulin basally (Figure S1). Transcript levels of glucagon, the endocrine hormone normally expressed in alpha cells, were unaffected by the compound. Expression of *Pdx1*, a beta cell-specific transcription factor capable of directly activating the *Ins2* promoter [23], was significantly induced at 1.65 µM and higher. An examination of the time course of gene expression revealed that GW8510 induced *Ins2* gene expression to its maximum after 48 hours, while Pdx1 was only up-regulated about two-fold after 96 hours (Figure 1B).

Because BRD7389 treatment enhanced insulin secretion in dissociated human islet cells [12], we decided to explore the effects of GW8510 on the same process. Five-day treatment with GW8510 increased basal insulin secretion at 1.67 mM glucose as well as glucose-stimulated insulin secretion at 16.7 mM (Figure 1C). Interestingly, treatment with low levels of staurosporine, a potent broad-range kinase inhibitor [24], also enhanced insulin secretion in this system. Examination of the total cell number and the numbers of alpha and beta cells, quantified by immunofluorescence analysis, revealed that GW8510 treatment decreased the total number of cells, but did not cause a significant decline in beta cell numbers (Figure 1D, E). Interestingly, the number of alpha cells seems to be increased following treatment with both 3.3 µM GW8510 and staurosporine (Figure 1D, E). Pancreatic islets are made up of a heterogeneous population of cells [2], and it is difficult to pinpoint a compound's effect on a particular cell type, even in the dissociated islet system. Therefore, we decided to focus on elucidating the mechanism of GW8510-induced insulin expression in mouse alpha cells.

**Figure 2:**
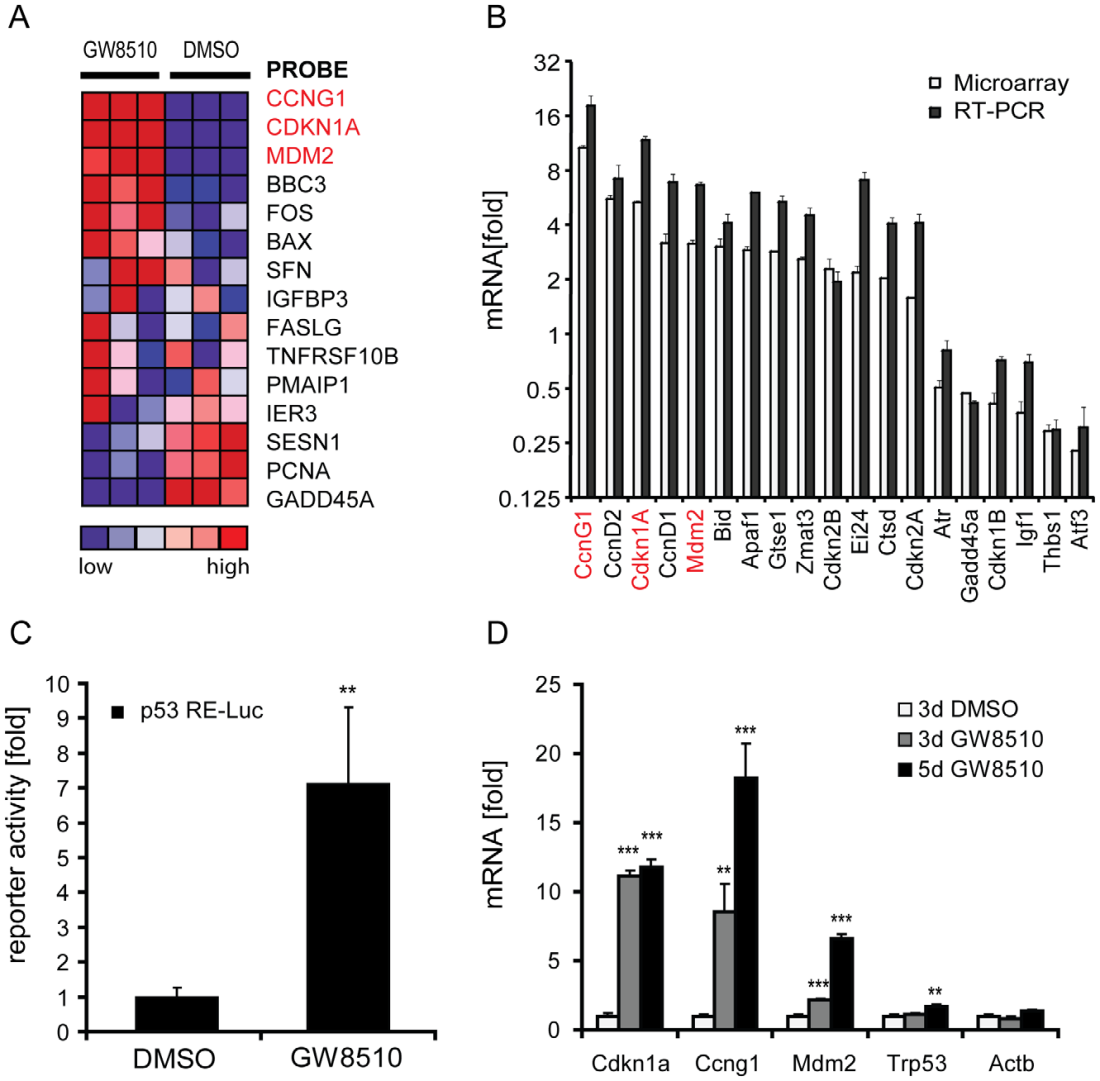
Involvement of the p53 pathway and quantification of p53 transcriptional activity and expression levels of p53 target genes following GW8510 treatment of alpha cells. (A) Heat-map display of one of the enriched gene sets (INGA_p53_TARGETS) in gene-expression profiling of alpha cells treated with 3.3 µM GW8510 for five days. Relative expression values in three biological replicates are plotted by color. Red, high expression levels, blue, low expression levels. (B) Reproducibility of gene-expression changes following GW8510 treatment, measured by microarray and qPCR. (C) Cellular p53 activity measured using a dual-luciferase reporter system. Activity of the firefly luciferase p53-reporter construct was normalized to constitutively active co-transfected *Renilla* luciferase, and to positive and negative controls. (D) qPCR measurement of transcript levels of direct p53 targets following 3- and 5-day treatments with 1.65 µM GW8510. Data represent the mean±SD of at least three experiments; **p<0.01 and ***p<0.001.

Since the induction of *Ins2* gene expression precedes *Pdx1* in this case, the initial increase in *Ins2* expression is likely to be induced by a mechanism not involving *Pdx1*. Furthermore, knock-down of CDK2 (Figure S2A) only marginally increased *Ins2* expression, while the use of other known CDK2 inhibitors (Figure S2B) had no effect on *Ins2* expression, suggesting that mechanisms other than CDK2 inhibition are likely to be responsible for up-regulation of *Ins2* by GW8510. Thus, in order to determine a potential mechanism of insulin induction, we treated mouse alpha cells with 3.3 µM GW8510 or 0.1% DMSO for five days, and performed gene-expression profiling of nearly 14,000 transcripts (see Methods). Following GW8510 treatment, 364 genes were up-regulated and 347 genes were down-regulated by at least two-fold over the matched vehicle controls (Figure S3). Gene-set enrichment analysis (GSEA) [25] revealed that gene sets containing p53-responsive genes were significantly enriched after GW8510 treatment (Table S1). Specifically, microarray measurements showed that the p53 transcriptional targets *Cdkn1a*, *Mdm2*, and *Ccng1* were up-regulated by compound treatment (Figure 2A). Microarray-detected transcriptional changes in cell cycle-specific genes and direct p53 transcriptional targets were confirmed on the same samples by quantitative real-time RT-PCR (Figure 2B).

Seeing as p53-responsive genes were induced following compound treatment, we sought to determine whether p53 transcriptional activity itself was enhanced. Using a p53-luciferase reporter-gene assay, we found that 24-hour treatment with 3.3 µM GW8510 increased reporter activity approximately seven-fold over DMSO-treated controls (Figure 2C). Quantitative real-time RT-PCR measurements confirmed that the transcript levels of *Cdkn1a*, *Mdm2*, and *Ccng1* were also significantly up-regulated following treatment with 1.65 µM GW8510 (Figure 2D). In particular, *Cdkn1a* and *Ccng1* were strongly induced after both three- and five-day treatments. *Mdm2* transcript was only slightly affected by three-day treatment, but showed significant up-regulation after five days with 1.65 µM GW8510. This GW8510 concentration was used in all further studies, because we could detect a full effect on *Ins2* induction without the toxicities observed at 3.3 µM.

**Figure 3:**
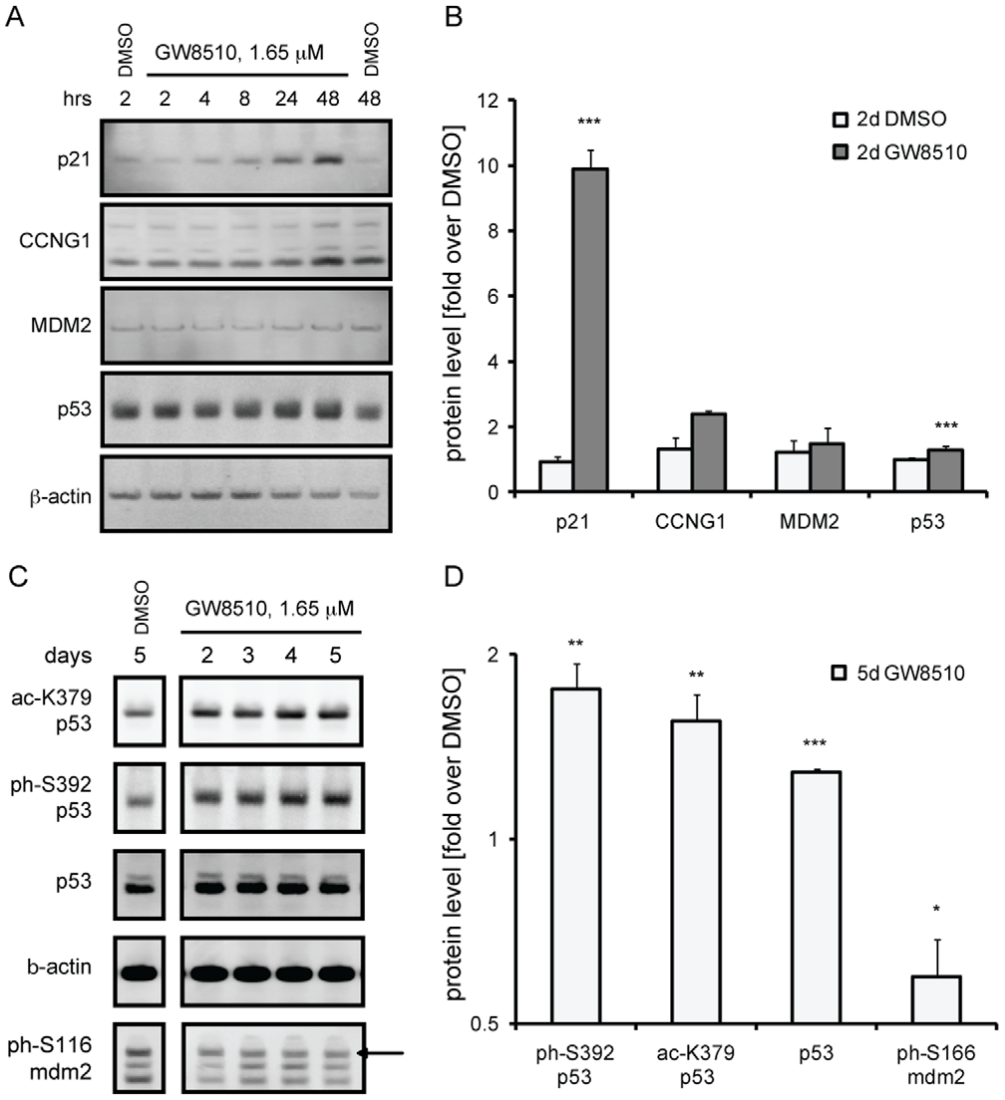
GW8510 treatment effects on protein levels of p53 transcriptional targets and on post-translational modification status of p53 and MDM2. (A) Western blot analysis and (B) quantification of protein levels of direct p53 targets following a two-day time-course with 1.65 µM GW8510. (C) Western blot analysis and (D) quantification of total protein and post-translational modification levels following 2–5 days of treatment with 1.65 µM GW8510. Data represent the mean ± SD of 3 biological replicates; *p<0.01, **p<0.01 and ***p<0.001.

Next, we explored downstream effects of p53 activation by GW8510 on protein levels in alpha cells. The protein product of *Cdkn1a*, p21, was increased after as little as eight hours, and elevated more than ten-fold over basal levels at 48 hours (Figure 3A, B). Cyclin G protein production was also increased, but the induction was slower and reached only a two-fold increase over basal levels by 48 hours of treatment. As with other cyclins, cyclin G is an unstable protein that is quickly degraded [26], but we could detect up-regulation of both the full-length protein and the degradation product. Consistent with our previous assessment of early *Mdm2* expression, MDM2 protein levels were unaffected after 48 hours of treatment with GW8510.

Cellular p53 activity is regulated at the post-translational level by combinations of modifications, such as phosphorylation and acetylation. For example, Ser392 phosphorylation has been reported to increase p53 DNA-binding capacity and transcriptional activity [27], [28]. Furthermore, p53 acetylation promotes p53 stability and accumulation [29]. Since we observed an increase in p53 activity after treatment with GW8510, we decide to examine p53 post-translational modification status. We observed that two- to five-day treatment with 1.65 µM GW8510 increased Ser392 phosphorylation in alpha cells, indicating a higher transactivation capacity of p53 following compound treatment (Figure 3C,D). We also detected an up-regulation of K379 acetylation (Figure 3C, D), which is also consistent with increased p53 activity. MDM2, a direct p53 transcriptional target, is an E3 ubiquitin ligase that regulates p53 stability by targeting it for degradation through a negative feedback mechanism [20]. Akt-mediated phosphorylation of MDM2 on Ser166 increases its nuclear localization and interaction with p300, which in turn, enhances p53 ubiquitination and degradation [30], [31]. Treatment with GW8510 caused a decrease in Ser166 phosphorylation (Figure 3C, D), presumably preventing p53 ubiquitination and enhancing p53 stability. Hence, the post-translational modification states of p53 and MDM2 are consistent with enhanced cellular p53 activity, and, therefore, with previous results indicating up-regulation of p53 transcriptional targets following compound treatment.

**Figure 4:**
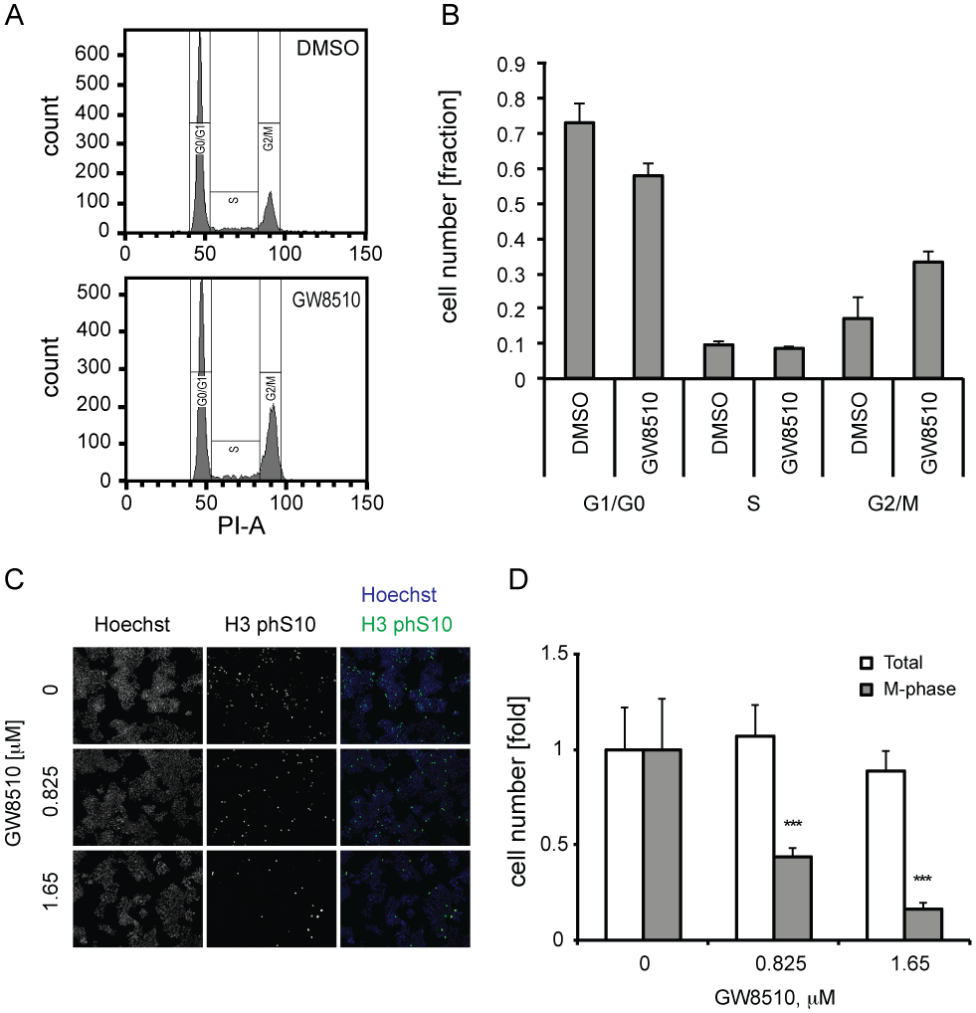
Cell-cycle effects of GW8510 treatment. (A) FACS-generated histograms of propidium iodide stained cells treated with either vehicle control or GW8510 for 3 days. (B) Quantification of cell-cycle distributions from gated cellular populations in A expressed as percentage of the total cellular population. Data represent the mean ± SD of two biological replicates. (C) and (D) M-phase immunofluorescence analysis and quantification using histone H3 phospho-Ser10 as a mitosis marker. Total cells were counted using Hoechst nuclear stain. Representative images are shown for Hoechst, histone H3 phospho-Ser10, and overlay at indicated GW8510 concentrations. Values are expressed as fold over vehicle-treated controls. Data represent the mean ± SD of at least 3 biological replicates; ***p<0.001.

Because GW8510 is reported to inhibit CDK2 [13] and has been shown here to enhance p53 activity, we examined the cell-cycle profile of alpha cells following compound treatment. CDK2 inhibition leads to an arrest in G1/S, while p53 over-expression and activation can induce either a G1/S or a G2/M cell-cycle arrest [32]. In light of our results showing elevated p21, cyclin G, and MDM2 levels, we anticipated that compound treatment would produce a cell-cycle phenotype. Indeed, FACS analysis following three-day treatment with GW8510 showed an enrichment of the G2/M population, from 17 to 33% (Figure 4A, B). This result suggested a G2/M arrest following induction of p53 activity, as opposed to G1/S arrest, which should result in enrichment of the G0/G1 population. We observed a decrease in the number of mitotic nuclei following treatment with GW8510 (Figure S4A), suggesting a delayed entry into mitosis. In contrast, the DNA-damaging agents doxorubicin and etoposide caused an increase in the number of mitotic nuclei (Figure S4A), consistent with previous observations that DNA damage delays exit from mitosis [33]. We also observed that GW8510 had no effect on phosphorylation of either ATR (Figure S4B) or ATM (Figure S5) protein kinases, which are activated following DNA damage [34]. In contrast, doxorubicin and etoposide increased ATR phosphorylation (Figure S4B) and activated the ATM-CHK2-p53 pathway (Figure S5). Accordingly, these DNA damaging agents did not induce insulin expression in alpha cells (Figure S6). We sought further evidence of a G2/M arrest by immunofluorescence analysis for Ser10 phosphorylation of histone H3, a marker of mitosis [35]. GW8510 treatment decreased the proportion of mitotic cells in a concentration-dependent manner (Figure 4C, D). The increase in the G2/M population, and the decrease in the number M-phase cells, indicate enrichment of G2, as would happen following a G2/M arrest.

**Figure 5:**
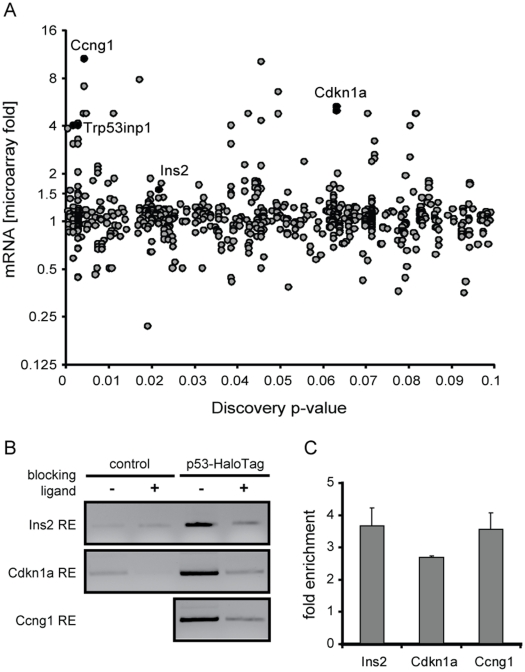
Evaluation of p53 response elements. (A) cisRED prediction of p53 response elements in promoter regions of indicated mouse genes. Discovery *p*-value is plotted against experimentally determined fold change in gene expression. Selected genes are highlighted. (B) ChIP-PCR analysis of predicted p53-response elements in promoter regions of *Ins2*, *Cdkn1a* and *Ccng1*. Cells were either untransfected (“control”) or transfected with recombinant tagged p53 (“p53-HaloTag”), and p53-bound DNA immunoprecipitated. PCR was then performed on Ins2, Cdkn1a, or Ccng1 regions containing predicted p53-response elements. The presence of blocking ligand helps determine the specificity of the interaction. (C) Fold enrichment of promoter binding is calculated over the corresponding blocking ligand control in the p53-HaloTag condition.

Since p53 is a transcription factor, the trans-activation capacity of which seems to be enhanced following treatment with GW8510, we sought to determine whether p53 could directly trans-activate Ins2. We mined a database for genome-scale computational discovery of conserved regulatory elements, cisRED, in the mouse genome, which contains conserved sequence motifs in promoters of about 17,500 genes [36]. Interestingly, cisRED analysis predicted a p53 response element in the promoter region of the *Ins2* gene, with a discovery *p*-value of 0.02 (Figure 5A, Table S2). This analysis also predicted known response elements in p53 target genes, such as *Cdkn1a* and *Ccng1*, with discovery *p*-values of 0.06 and 0.004, respectively. It should also be noted that no such response element is predicted for Ins1, and no Ins1 up-regulation was, in fact, detected by microarray or real time RT-PCR (data not shown).

We verified the presence of p53-response elements in the *Ins2* gene by using a covalent chromatin-capture protocol with recombinant tagged p53 in mouse alpha cells. This method employs covalent bond formation between the tag and the ligand immobilized on beads, and has the advantage of being more efficient and robust than conventional antibody-based ChIP. The affinity tag in this system, haloalkane dehalogenase, is a derivative of a bacterial hydrolase that allows covalent site-specific tethering to resin (see Methods). Since the resin contains immobilized synthetic ligands, the excess of free ligand can block the interaction between the tagged protein and the resin. Thus, addition of blocking ligand to half of the reaction allows quantification of enrichment over the corresponding control [37]. PCR analysis following p53 ChIP revealed the presence of predicted response elements in promoter regions of known p53 transcriptional targets, *Cdkn1a* and *Ccng1*, as well as *Ins2* (Figure 5B, C). Electrophoretic analysis and quantification of PCR products demonstrated that 1.34% of total input DNA is pulled down by p53 at the *Cdkn1a* promoter and 0.15% at the *Ins2* promoter (Figure S7).

**Figure 6:**
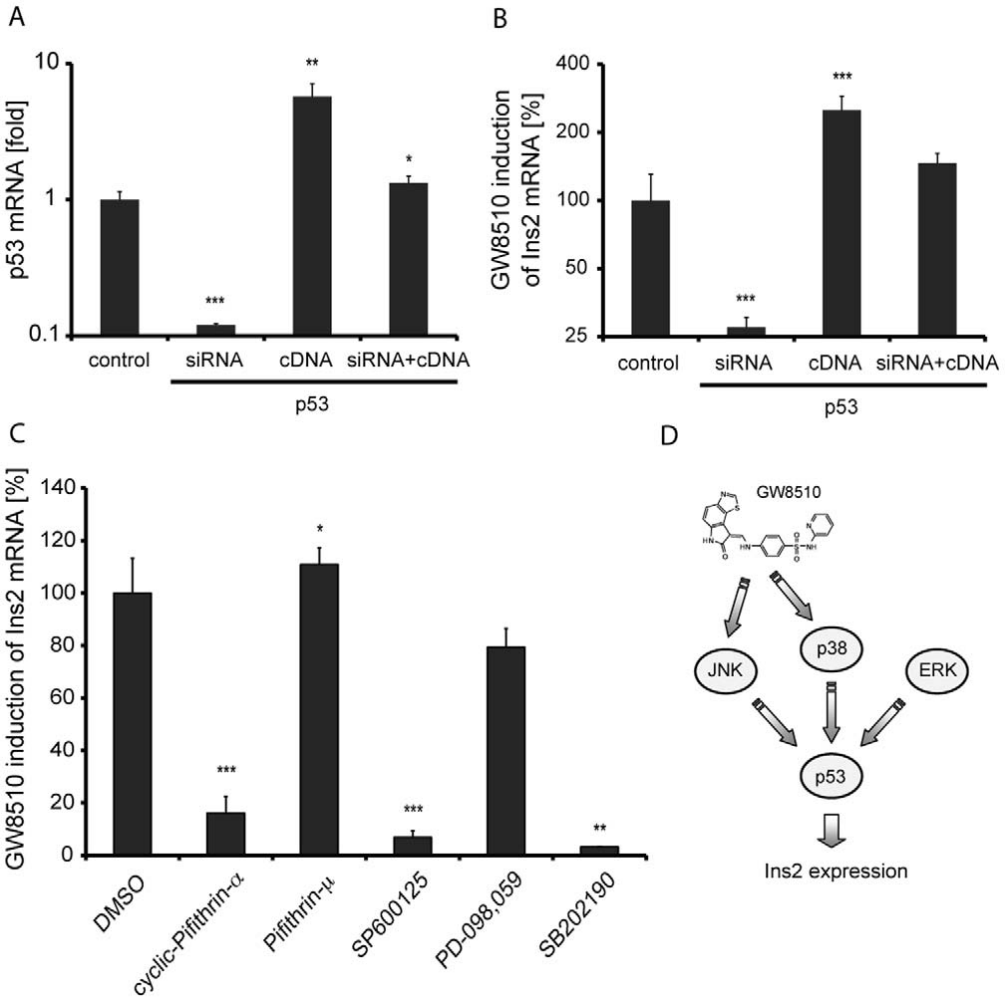
Effects of manipulation of p53 levels and activity on induction of *Ins2* by GW8510 treatments. (A) Experimental knockdown and over-expression of p53 in alpha cells and (B) its effects on *Ins2* induction by 3-day treatment with 1.65 µM GW8510. (C) Co-treatment with small molecule inhibitors of p53 and upstream targets in the p53 signalling pathway and their effect on *Ins2* induction following treatment with GW8510. (D) Proposed model for GW8510-mediated induction of insulin expression via activation of p53 transcriptional activity. All data represent the mean±SD of at least three experiments; *p<0.05, **p<0.01 and ***p<0.001.

We then determined whether up-regulation of p53 transcriptional activity was related to *Ins2* induction by GW8510, and whether modulation of p53 levels or activity would have an impact on induction of *Ins2* expression. P53 levels in alpha cells were manipulated by overexpression or by siRNA-mediated knock-down (Figure 6A). Knock-down of p53 decreased the induction of *Ins2* by GW8510 by 75%, while p53 overexpression resulted in a three-fold increase in *Ins2* induction following three-day treatment with 1.65 µM GW8510 (Figure 6B). We also modulated p53 activity using chemical probes that either target p53 directly or act upstream in the signal transduction pathways leading to p53 activation. Pifithrin-α, a reversible inhibitor of p53-mediated apoptosis and p53-dependent transcription [38], suppressed GW8510-induced *Ins2* expression by 80% (Figure 6C). Pifithrin-μ, which inhibits p53 mitochondrial signalling pathway without having an effect on p53 transcriptional trans-activation capacity [39], had no effect on induction of *Ins2* by GW8510 (Figure 6C). We examined pathways upstream of p53, and observed that both SP600125, a JNK1/2/3 inhibitor [40], and SB202190, a p38 kinase inhibitor [41], almost entirely suppressed *Ins2* induction by GW8510 (Figure 6C). In contrast, the ERK inhibitor PD-09859 [42] reduced GW8510-induced expression by only 20% (Figure 6C). We confirmed that siRNA-mediated silencing of JNK and p38 also reduced GW8510-induced insulin expression (Figure S8). This chemical epistasis analysis indicates that GW8510 does not likely act at the level of p53 directly, but rather upstream of p53, in signalling pathways involving JNK and p38 (Figure 6D).

## Discussion

Small molecule-mediated alterations in cell state are important in diseases of cellular deficiency such as type-1 diabetes. An increase in insulin expression in other pancreatic cell types could be a valuable part of a strategy to increase beta-cell mass. We report induction of insulin expression in murine pancreatic alpha cells with GW8510, a small molecule annotated as a CDK2 inhibitor. We previously described a putative kinase inhibitor, BRD7389, able to modulate insulin levels in mouse alpha cells [12], but have found that GW8510 more strongly increases *Ins2* mRNA levels. Interestingly, both compounds enhanced insulin secretion in dissociated human islets. We show that in the donor tested, treatment with GW8510 and a general kinase inhibitor, staurosporine, potentiated both basal and the glucose-stimulated insulin secretion. In addition, a previous report indicates that wortmannin, a phosphatidylinositol 3-kinase inhibitor, augmented insulin secretion at 15 mM glucose [43]. Currently available methodology does not enable us to distinguish between the effects on insulin release from beta cells, which represent the majority of the islet endocrine cell population, and potential contributions from other islet cell types. However, these findings justify further exploration of kinase inhibition on endocrine-cell composition of pancreatic islets, generation of insulin in non-beta cell types, and beta-cell function.

We then explored the mechanism of GW8510-induced insulin expression in mouse alpha cells. The canonical regulator of insulin expression, *Pdx1*, was not implicated in the initial burst of *Ins2* induction. In an attempt to identify the underlying mechanism of action, we examined the effects of GW8510 on the alpha-cell transcriptome, which revealed involvement of p53 pathway activation in the observed phenotype. Furthermore, we validated a cisRED-predicted p53 response element in the *Ins2* promoter region. Following identification of p53 as a direct transcriptional regulator of *Ins2* expression, we demonstrated that increasing p53 levels and activity enhances compound-mediated insulin induction, while decreasing p53 suppresses these effects.

P53 is present at low levels in normal tissues, including the pancreas. However, a comparison of expression levels across rodent pancreatic cell types revealed an enrichment of p53 expression in alpha and beta cells compared to intact pancreatic islets or to whole pancreas preparations [44]. We observed high p53 protein levels in the alpha cell line, αTC1.6 (Figure 2C), indicating that modulating p53 activity in alpha cells could be a feasible strategy to induce insulin expression. Furthermore, treatment of NIH3T3 mouse embryonic fibroblasts with GW8510 enhanced expression of the canonical p53 transcriptional targets, e.g. *Cdkn1a* and *Ccng1*, but did not show an effect on *Ins2* mRNA levels (Figure S9). Hence, the p53-dependent insulin induction by GW8510, observed in pancreatic alpha cells, depends on the phenotypic cellular context and does not occur in a non-pancreatic cell type.

Consistent with previous observations in a variety of cell lines [45], we found that p53 binds to response elements in promoters of its target genes under basal conditions (Figure 5). DNA binding itself, however, does not cause expression of p53 targets [45]; execution of the p53 transcriptional program depends on the necessary set of post-translational modifications and interactions with appropriate co-regulators. Increasing p53 levels by overexpression, or stimulating its activity with inhibitors of p53-MDM2 interaction, did not achieve the same effect (data not shown), suggesting that intervention upstream of p53 is necessary for the observed phenotype.

GW8510 does not appear to act at the level of p53 directly, but acts upstream of p53 to target JNK- and p38-dependent signal-transduction pathways. Chemical (Figure 6C) or genetic (Figure S8) inhibition of either JNK or p38 activity interfered with induction of *Ins2* by GW8510. JNK and p38 kinases directly phosphorylate and trans-activate p53 under stress conditions [46], including genotoxic stress and DNA damage [47]. However, treatment with the DNA damaging agents doxorubicin and etoposide did not induce insulin expression in alpha cells (Figure S6), suggesting that GW8510 does not function by inducing DNA damage. The suppression of GW8510's effects by JNK and p38 kinase inhibitors indicates that GW8510 acts upstream of these two kinases, and that both signal-transduction pathways are required to achieve the p53 post-translational modification necessary for transcriptional activation.

Historically, p53-mediated transcription of target genes has been thought to occur through sequence-specific binding of the p53 tetramer to the consensus response element, composed of two decamer half-sites separated by a 0–13 bp spacer region [48]. The breadth of p53 transcriptional targets has been recently expanded following identification of functional non-canonical response elements, like the ¾ and the ½ sites [49]. The predicted 11-bp p53 half-site response element is located 1,267 bps upstream of TSS for the mouse *Ins2* gene, well outside the *cis*-regulatory promoter-proximal region, which extends ∼300 bp upstream and 100 bp downstream of the TSS [8]. The compact nature of the promoter and the combinatorial complexity of transcription factor regulatory elements allow tight positional and temporal control of insulin expression in beta cells [8]. However, more distal regulatory sequences may perhaps be utilized to induce ectopic insulin expression in a non-beta cell type. Additionally, a distal regulatory element may be in spatial proximity with the target gene or other regulatory factors at the proximal promoter region through looped chromatin conformation [50]. A detailed exploration of looped genomic interactions at the insulin promoter may yield further insights into regulation of insulin expression in pancreatic beta cells and the potential for up-regulating insulin expression in a non-beta cell type.

These results show that small-molecule activation of p53 in a JNK- and p38-dependent manner can regulate *Ins2* gene expression in mouse alpha cells. The cisRED database also predicts an identical response element located 1,444 bp upstream of the rat *Ins2* TSS (Table S5), indicating similarity in the regulation of mouse and rat *Ins2* genes. In contrast, there is >65% identity in the main regulatory region of the insulin promoters of humans and non-primate mammals (−300 to +1), and quickly drops to <40% identity in the first 600 bp upstream of the TSS [8]. Nevertheless, the database also predicts a sequence orthologous to the p53 response element in the human insulin gene, located 202 bp downstream of the TSS (Table S5). Evaluation of this prediction will help determine whether modulation of p53 activity could also be used to alter insulin expression in human endocrine cell types.

## Materials and Methods

### Reagents

All chemicals were obtained from Sigma Aldrich. Mouse pancreatic alpha cell line αTC1 (clones 6 and 9) was purchased from the American Type Culture Collection. Primers were bought from Origene and Eurofins MWG Operon. Trp53, cdk2, p38 and JNK siRNA constructs were purchased from Applied Biosciences, mouse p53 vector was obtained from Origene (MC205636), and p53-luciferase reporter constructs were bought from SABiosciences. Antibodies used in this study were purchased from Cell Signaling for p53 (2524), p53 phospho-S392 (9281), p53 acetyl-K379 (2570), mdm2 phospho-S116 (3521), and histone H3 phospho-Ser10 (9706), Santa Cruz for p21 (sc-6246), cyclin G1/G2 (sc-851), and mdm2 (sc-56155), and Sigma for β-actin (A1978). Fluorescently-labelled secondary antibodies were purchased from Jackson ImmunoResearch, and poly-HRP conjugated antibodies were purchased from Thermo Scientific Pierce.

### Cell culture and compound treatments

αTC1 cells were grown in DMEM containing 1 g/L glucose, supplemented with 10% FBS, 50 U/mL penicillin and 50 U/mL streptomycin. Pancreatic islets from one donor (Age: 47 BMI: 23, purity: 85%, viability: 99%) were dissociated and cultured as previously described [12]. For compound treatments, cells were plated in 6-well plates for Western blot and FACS analysis, 24-well plates for gene-expression analysis and 96-well plates for immunofluorescence analysis. Cells were allowed to adhere overnight before addition of compound, and for 5-day treatments, media was changed and new compound added on day 3. All compound treatments were performed in 0.1% final DMSO concentration.

### Gene expression measurements

Following compound treatment, cells were lysed and RNA isolated using the RNeasy Plus Mini kit (Qiagen) according to the manufacturer's protocol. 500 ng of RNA was reversed transcribed using High Capacity RNA-to-cDNA Master Mix (Applied Biosystems). Quantitative PCR was performed with SYBR Green PCR Master Mix (Applied Biosystems) on an Applied Biosystems 7900HT real-time PCR machine using the primers listed in Table S3. Microarray analysis was performed by the Broad Institute Genetic Analysis Platform on 500 ng of total RNA using GeneChip Mouse Genome 430A arrays from Affymetrix, measuring about 22,000 transcripts for approximately 14,000 genes. All data is MIAME compliant, with the raw data deposited in Gene Expression Omnibus (GEO), accession number GSE31102.

### Western blot analysis

Cell extracts were generated by lysing cells in modified RIPA buffer containing 1% NP-40, 0.1% sodium deoxycholate, 150 mM NaCl, 1 mM EDTA, 50 mM Tris, pH 7.5 supplemented with protease inhibitors (Roche) and phosphatase inhibitors (1% v/v cocktail 1, 0.5% v/v cocktail 2, 1% v/v cocktail 3, Sigma). Protein concentrations were measured using BCA Protein Assay Kit (Thermo Scientific Pierce), and 20 µg of each sample were run on E-Page 48 gels (Invitrogen) and transferred to PVDF membranes using an iBlot (Invitrogen). Membranes were probed with 1∶500 dilutions of primary antibodies from Cell Signaling, 1∶100 dilutions of primary antibodies from Santa Cruz, and 1∶1000 dilutions of secondary poly-HRP conjugated antibodies (Thermo Scientific Pierce). Blots were imaged on an Image Station 4000MM PRO (Kodak/Carestream), and band intensities were quantified using ImageJ software.

### Immunofluorescence measurements

15,000 αTC1 cells per well were plated in 50 µL media in black optical-bottom tissue culture-treated 96-well plates (Corning). Following compound treatment, cells were fixed with 4% paraformaldehyde for 20 minutes at room temperature. Cells were permeabilized in PBS supplemented with 0.3% Triton X-100 for 20 minutes at room temperature and blocked with 3% BSA in PBS supplemented with 0.1% Tween-20 (PBSTB3) for 30 minutes. Cells were then incubated in 1∶250 dilution of primary antibody in PBSTB3 overnight at 4°C. Following three washes with PBS, cells were incubated in secondary antibody and 10 µg/mL Hoechst 33342 in PBSTB3 for 1 h at room temperature in the dark. Following three washes with PBS, cells were imaged using an ImageXpress Micro automated microscope (Molecular Devices). Image analysis and quantification was performed using the “Cell Scoring” module of MetaXpress software (Molecular Devices).

### Transfections

siRNA transfections were performed as outlined in the Lipofectamine RNAiMax manufacturer's protocol (Invitrogen). 30 pmol total siRNA was transfected per well of a 24-well plate, using a combination of three siRNA constructs, 10 pmol each. Lipofectamine 2000 was used for vector transfections according to manufacturer's protocol (Invitrogen). 0.8 µg of DNA was transfected per well of a 24-well plate. Antibiotic-free DMEM supplemented with 10% FBS was used for all transfections, and was changed to the usual media 24 hours later. Indicated compound treatments were started during the media change.

### FACS analysis

Suspensions of αTC1 cells from a well of a 6-well plate (500 µl in PBS) were fixed in 5 mL of cold ethanol and left at 4°C overnight. Cells were washed twice and resuspended in 800 mL of PBS containing 1% BSA. Cells were stained by addition of 50 mL of 1 mg/mL propidium iodide solution (Invitrogen) and 100 mL of 10 mg/mL RNase A solution (Sigma), and incubated at 37°C for 30 minutes. Samples were analyzed on the BD LSRII flow cytometer.

### Reporter-gene assay

p53-luciferase constructs were resuspended in Opti-MEM and reverse-transfected into αTC1 cells using SureFECT transfection reagent according to manufacturer's protocol (SABiosciences). 24 hours following transfection, media was changed to DMEM containing 0.5% FBS and either 0.1% DMSO or the compound of interest. Dual-GLO luciferase assay was performed 24 hours after compound addition according to manufacturer's protocol (Promega). Firefly and *Renilla* luminescence signal were read consecutively on an EnVision Multilabel Plate Reader (PerkinElmer). Reporter signal in each experimental condition was normalized to the *Renilla* transfection control, and subsequently to positive- and negative-control reporter wells.

### Chromatin immunoprecipitation

Mouse p53 was amplified using the following primers:

Fw 5′-AAAAGCGATCGCCACTGCCATGGAGGAGTCACAGTC-3′

Rv 5′-AAAAGTTTAAACTCAGTCTGAGTCAGGCCCCA-3′

The insert was cloned into the pFN22K Halotag cmvd1 flexi vector using PmeI/SgfI restriction sites. Transfections were performed in 10-cm dishes overnight. Media was changed after 24 hours, and cells were fixed in 1% formaldehyde 48 hours after transfection. Samples were lysed in HaloCHIP lysis buffer and sonicated on ice using Branson Sonifier 250 Analog at 2.5 output with 6 cycles of alternating 10 seconds on and 10 seconds off. HaloCHIP system protocol (Promega) was performed on cell lysates according to manufacturer's instructions. Blocking ligand was added to half the sample. Primers for PCR analysis of putative p53 response elements are listed in Table S4.

## Supporting Information

Figure S1 **Effects of GW8510 on insulin expression in alphaTC1, clone 9 cell line.** Cells were treated for three days with the indicated concentration of GW8510, and mRNA collected for assessment of insulin expression by quantitative PCR. Gene expression was normalized to actin expression. Data represent the mean ± SD of three biological replicates; * *p*<0.05.
(TIF)

Figure S2 **Effects of reduction in cdk2 levels or activity on insulin expression in mouse alpha cells.** (**A**) siRNA-mediated silencing of cdk2 induces insulin gene expression approximately two-fold. Scrambled siRNA was used as a control. (**B**) *Ins2* gene expression changes following a 2-day treatment of αTC1 cells with cdk2 inhibitors at indicated concentrations. Data represent the mean ± SD of three biological replicates.
(TIF)

Figure S3 **Volcano plot of microarray measurements in alpha cells following five-day treatment with 3.3 µM GW8510.** Fold change is calculated over matched DMSO controls (n = 3) and plotted against the *p-value*. Genes down-regulated and up-regulated following GW8510 treatment at least 2-fold with p<0.01 are counted.
(TIF)

Figure S4 **Assessment of cell-cycle and ATR activation following treatment with GW8510 and known DNA-damaging agents.** (**A**) Percent mitotic nuclei induced by the indicated concentrations of each compound. (**B**) Phosphorylation of ATR was assessed by immunofluorescence, with the nuclear intensity of p-ATR staining, overlapping with Hoechst nuclear dye, quantified using MetaXpress (Molecular Devices). Data represent the mean±SD of at least three experiments; **p<0.05, **p<0.01 and ***p<0.001.
(TIF)

Figure S5 **Assessment of induction of the ATM pathway by GW8510.** Nuclear intensities of (**A**) phosphorylated ATM, (**B**) phosphorylated CHK2, and (**C**) phosphorylated p53 were assessed by immunofluorescence and analysis using MetaXpress software (Molecular Devices). Representative images are shown in (**D**). Data represent the mean±SD of at least three experiments; **p<0.05, **p<0.01 and ***p<0.001.
(TIF)

Figure S6 ***Ins2*** **gene expression changes following a 2-day treatment of aTC1 cells with DNA-damaging agents.** Cells were treated with (A) doxorubicin, (B) etoposide, (C) thymidine, and (D) ethidium bromide, at indicated concentrations. Data represent the mean±SD of three biological replicates.
(TIF)

Figure S7 **PCR analysis and quantification following halo-tag p53 ChIP of predicted p53-response elements in promoter regions of (A) a known p53 transcriptional target,** ***Cdkn1a*** **, and (B) the novel target,** ***Ins2*** **.** Percent input at each predicted response element is calculated from the standard input curve.
(TIF)

Figure S8 **Knock-down of JNK and p38 inhibit induction of Ins2 by GW8510.** Alpha cells were transfected with the indicated siRNAs for one day, followed by three-day treatment with 1.65 µM GW8510. mRNA was collected for analysis of *Ins2* gene expression by quantitative PCR, using actin as a normalization control.
(TIF)

Figure S9 **Effects of GW8510 on NIH3T3 cells.** Gene expression changes in NIH3T3 mouse embryonic fibroblasts following a 3-day treatment of aTC1 cells GW8510 at indicated concentrations. Data represent the mean±SD of three biological replicates; *p<0.05, **p<0.01 and ***p<0.001.
(TIF)

Table S1 **p53-pathway related GSEA gene sets found to be enriched following 5-day treatment of alpha cells with GW8510.**
(DOC)

Table S2 **CisRED prediction of p53 response elements (group 200034) in promoter regions of selected genes from the Mouse 4.0 database.**
(DOC)

Table S3 **Quantitative real-time PCR primers for indicated mouse genes.**
(DOC)

Table S4 **Primers used for PCR analysis of putative p53 response elements predicted by CisRED.**
(DOC)

Table S5 **Species comparisons of orthologous sequences of the p53 response element predicted in mouse Ins2 promoter region by CisRED.**
(DOC)
