# Correction: GW8510 Increases Insulin Expression in Pancreatic Alpha Cells through Activation of p53 Transcriptional Activity

**DOI:** 10.1371/annotation/c41ac082-32ac-4837-b4ed-9fd8ad91c1e0

**Published:** 2012-03-07

**Authors:** Dina Fomina-Yadlin, Stefan Kubicek, Amedeo Vetere, Kai Hui Hu He, Stuart L. Schreiber, Bridget K. Wagner

There was an error in the fourth author's name. The correct spelling is Kai Hui Hu He. The correct citation is: Fomina-Yadlin D, Kubicek S, Vetere A, Hu He KH, Schreiber SL, et al. (2012) GW8510 Increases Insulin Expression in Pancreatic Alpha Cells through Activation of p53 Transcriptional Activity. PLoS ONE 7(1): e28808. doi:10.1371/journal.pone.0028808. The correct author contributions are: Conceived and designed the experiments: DF-Y SK SLS BKW. Performed the experiments: DF-Y SK AV KHHH. Analyzed the data: DF-Y SK AV KHHH. Wrote the paper: DF-Y SK SLS BKW. 

