# Supplementary figures and images for: GW8510 Increases Insulin Expression in Pancreatic Alpha Cells through Activation of p53 Transcriptional Activity
Source: PLoS One. 2012 Jan 5;7(1):e28808. doi: 10.1371/journal.pone.0028808 (PMC3252286; doi:10.1371/journal.pone.0028808)

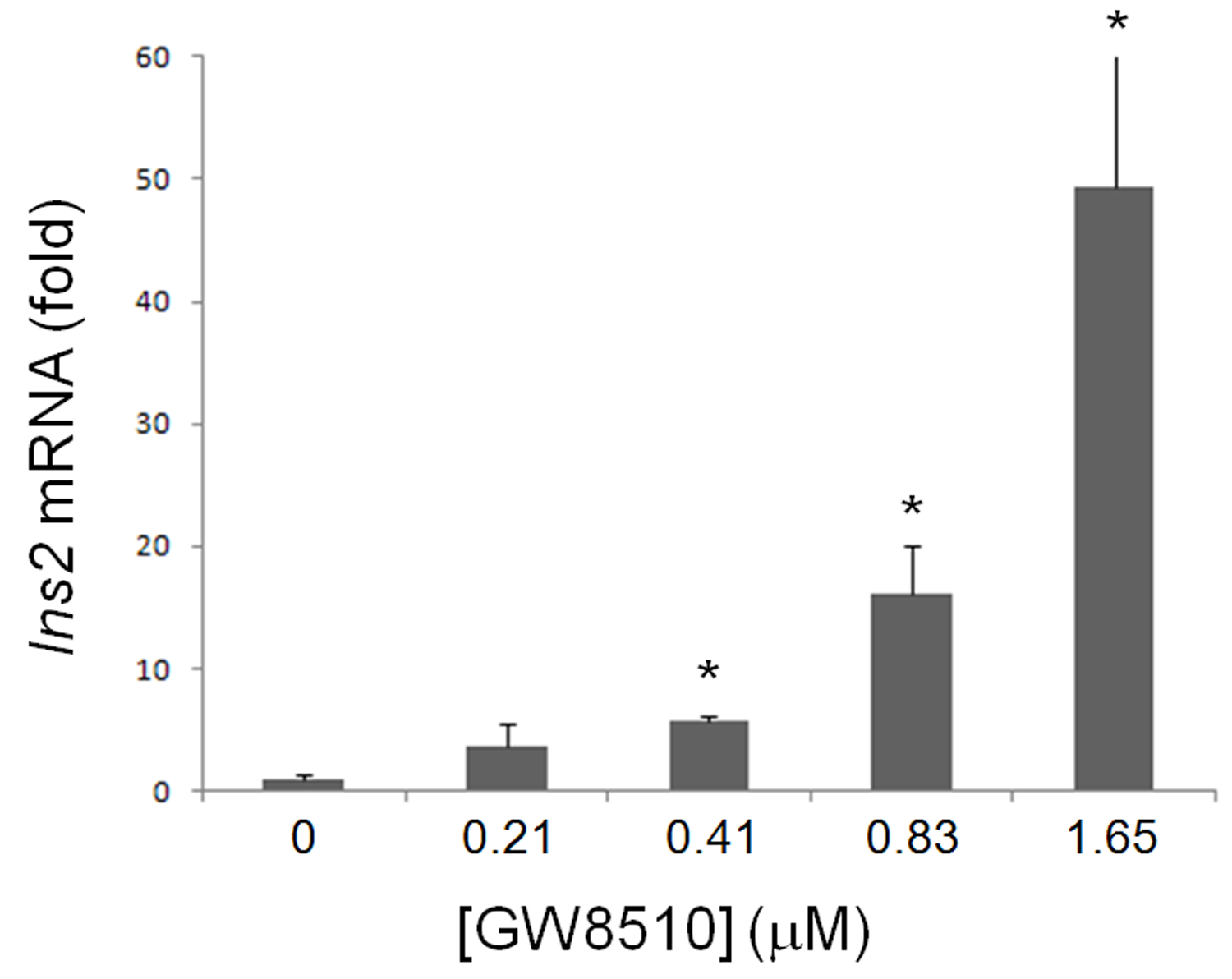

Supplement: Figure S1 — Effects of GW8510 on insulin expression in alphaTC1, clone 9 cell line. Cells were treated for three days with the indicated concentration of GW8510, and mRNA collected for assessment of insulin expression by quantitative PCR. Gene expression was normalized to actin expression. Data represent the mean ± SD of three biological replicates; * p<0.05. (TIF) [file pone.0028808.s001.tif]

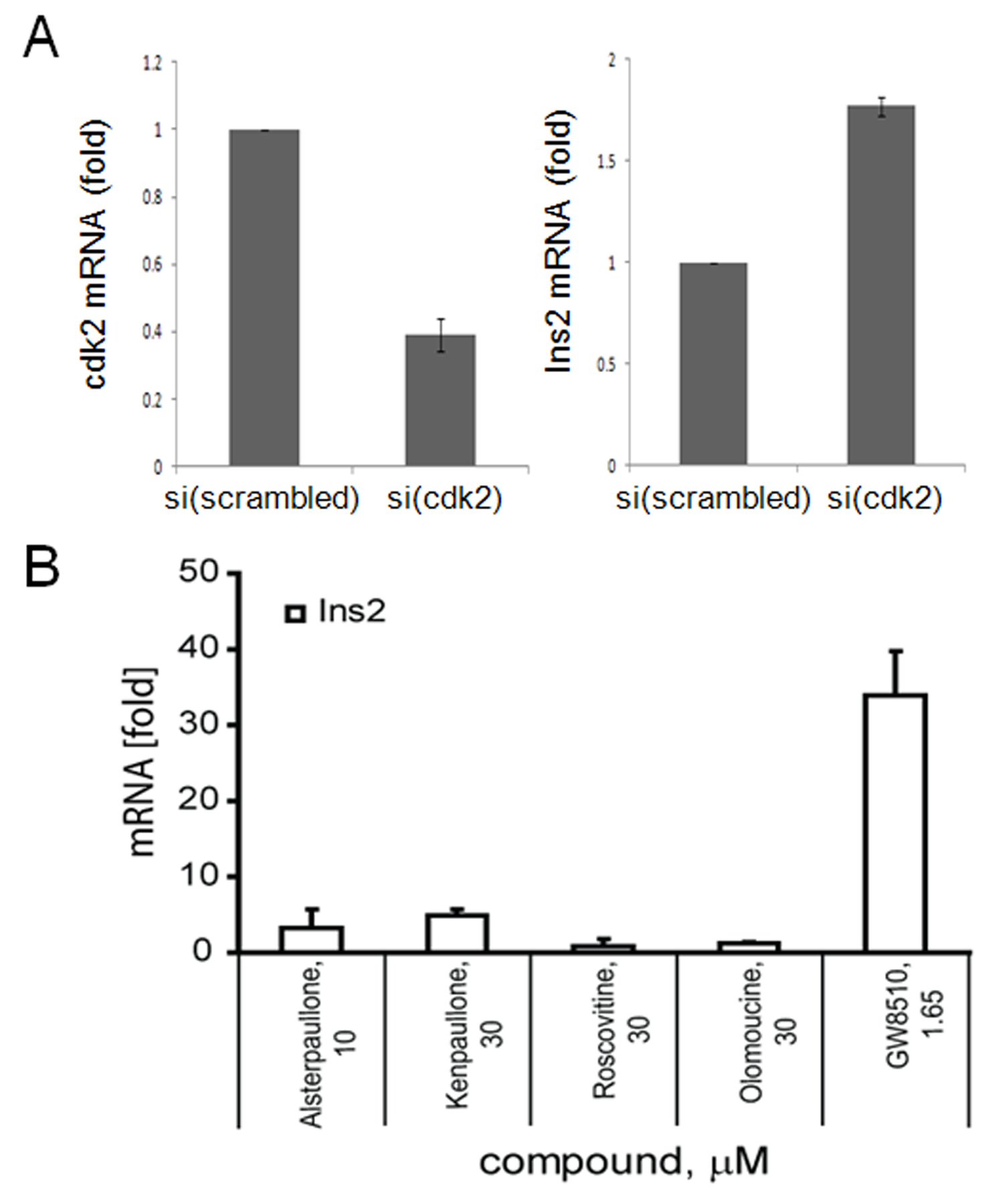

Supplement: Figure S2 — Effects of reduction in cdk2 levels or activity on insulin expression in mouse alpha cells. (A) siRNA-mediated silencing of cdk2 induces insulin gene expression approximately two-fold. Scrambled siRNA was used as a control. (B) Ins2 gene expression changes following a 2-day treatment of αTC1 cells with cdk2 inhibitors at indicated concentrations. Data represent the mean ± SD of three biological replicates. (TIF) [file pone.0028808.s002.tif]

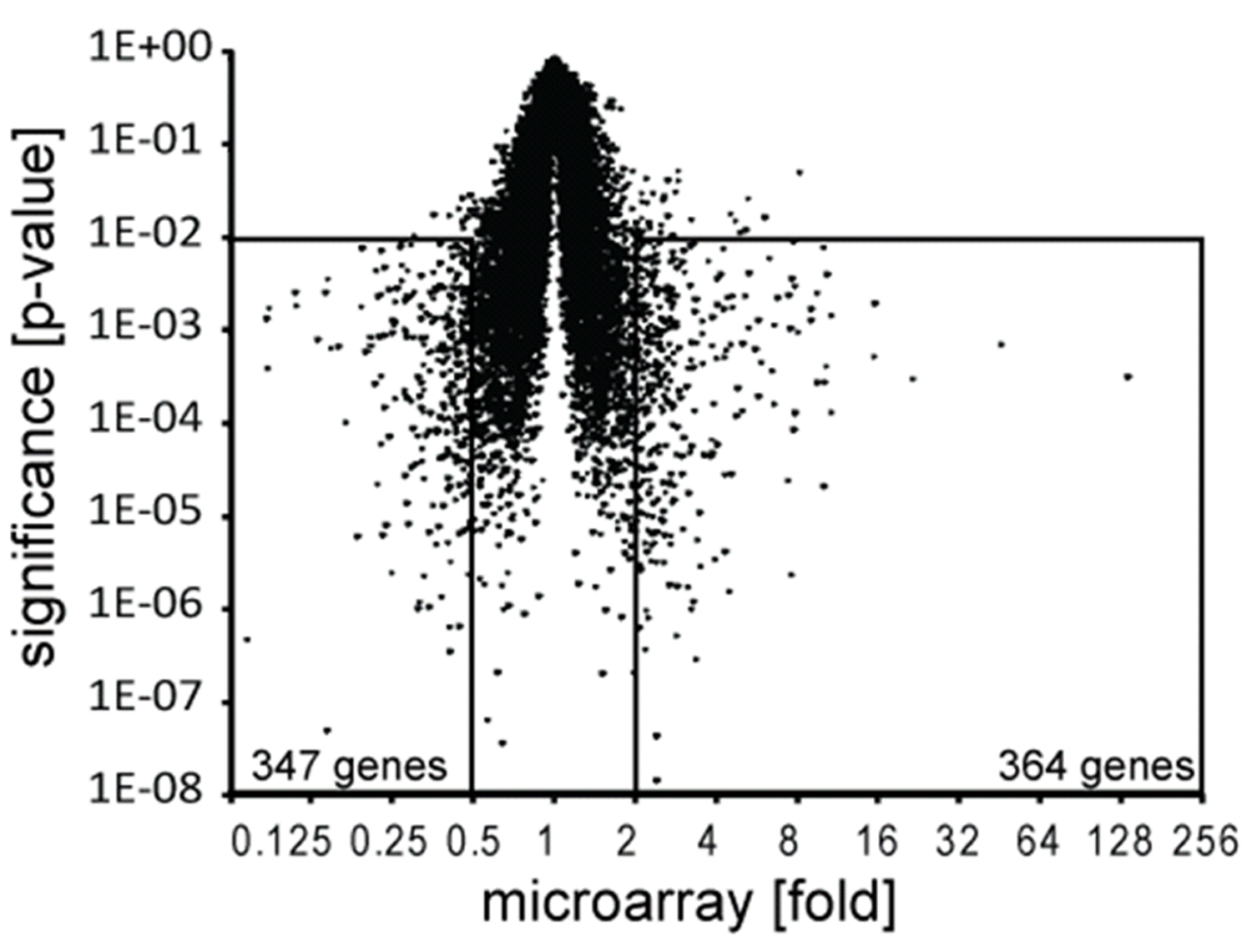

Supplement: Figure S3 — Volcano plot of microarray measurements in alpha cells following five-day treatment with 3.3 µM GW8510. Fold change is calculated over matched DMSO controls (n = 3) and plotted against the p-value. Genes down-regulated and up-regulated following GW8510 treatment at least 2-fold with p<0.01 are counted. (TIF) [file pone.0028808.s003.tif]

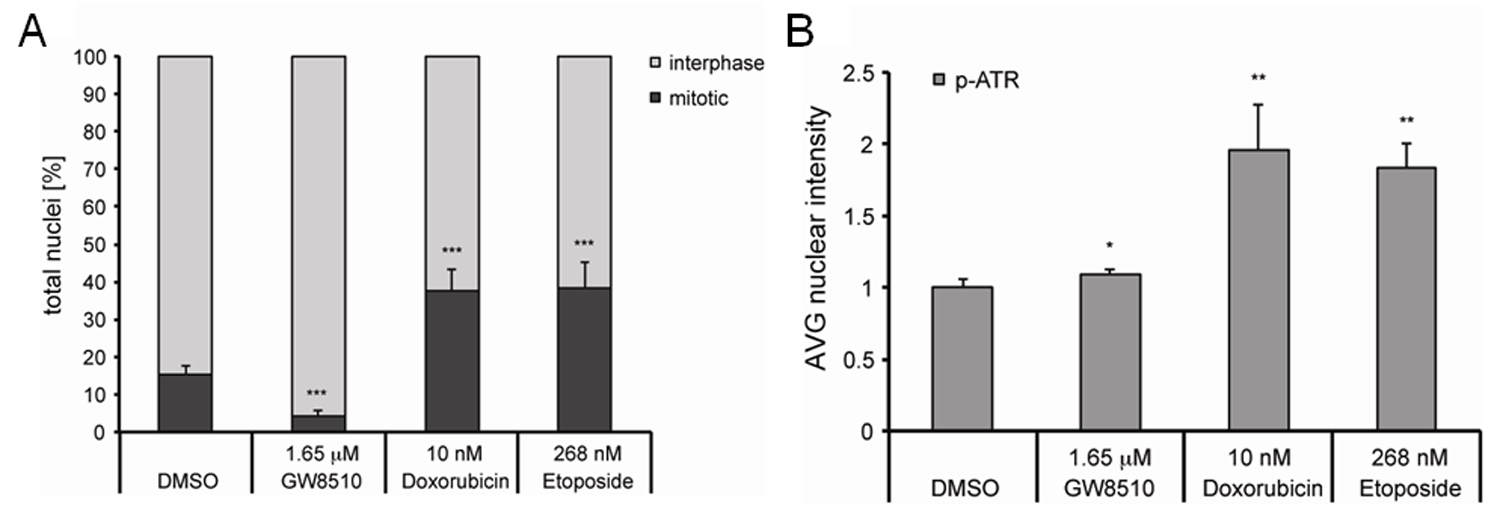

Supplement: Figure S4 — Assessment of cell-cycle and ATR activation following treatment with GW8510 and known DNA-damaging agents. (A) Percent mitotic nuclei induced by the indicated concentrations of each compound. (B) Phosphorylation of ATR was assessed by immunofluorescence, with the nuclear intensity of p-ATR staining, overlapping with Hoechst nuclear dye, quantified using MetaXpress (Molecular Devices). Data represent the mean±SD of at least three experiments; **p<0.05, **p<0.01 and ***p<0.001. (TIF) [file pone.0028808.s004.tif]

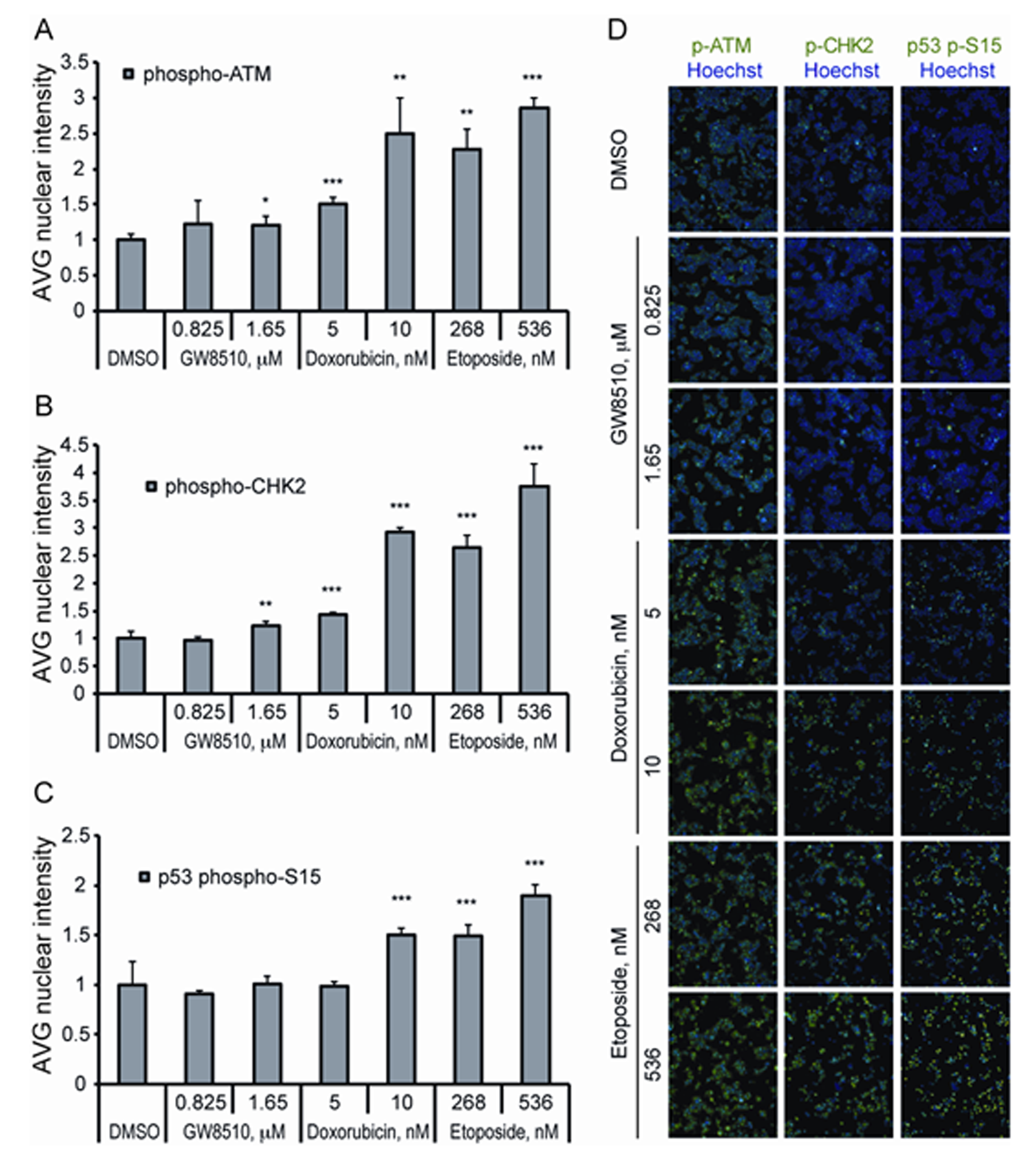

Supplement: Figure S5 — Assessment of induction of the ATM pathway by GW8510. Nuclear intensities of (A) phosphorylated ATM, (B) phosphorylated CHK2, and (C) phosphorylated p53 were assessed by immunofluorescence and analysis using MetaXpress software (Molecular Devices). Representative images are shown in (D). Data represent the mean±SD of at least three experiments; **p<0.05, **p<0.01 and ***p<0.001. (TIF) [file pone.0028808.s005.tif]

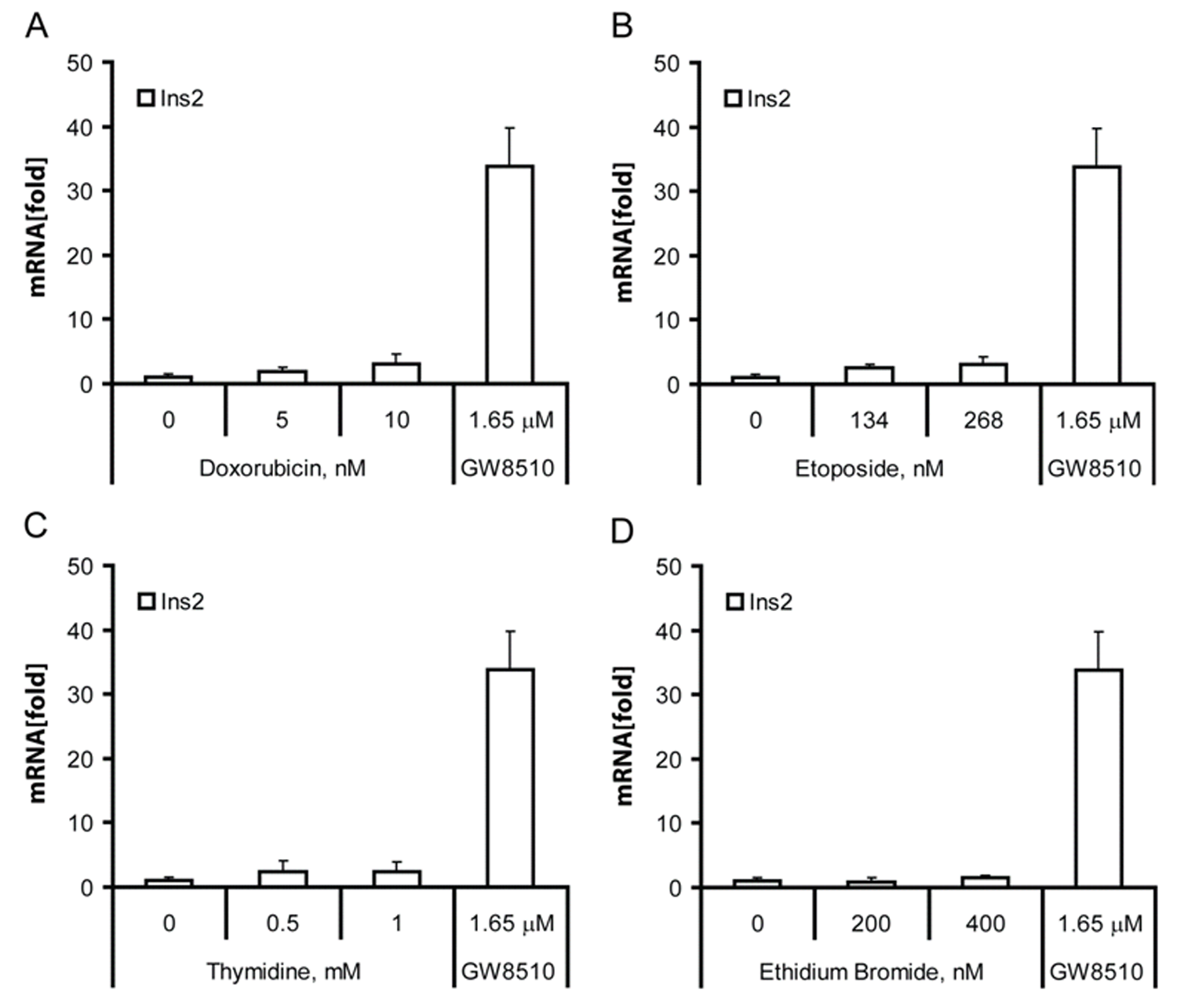

Supplement: Figure S6 — Ins2 gene expression changes following a 2-day treatment of aTC1 cells with DNA-damaging agents. Cells were treated with (A) doxorubicin, (B) etoposide, (C) thymidine, and (D) ethidium bromide, at indicated concentrations. Data represent the mean±SD of three biological replicates. (TIF) [file pone.0028808.s006.tif]

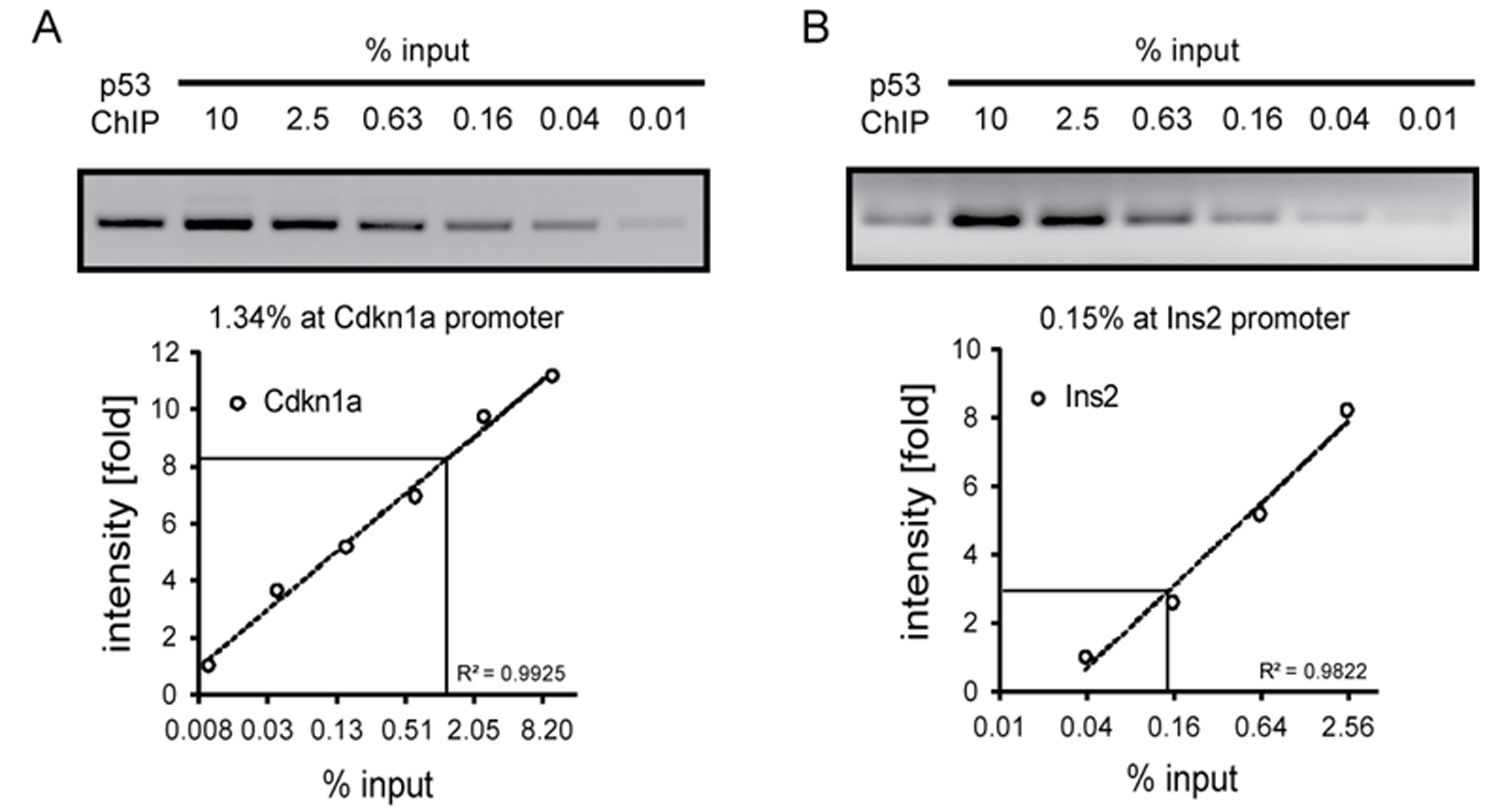

Supplement: Figure S7 — PCR analysis and quantification following halo-tag p53 ChIP of predicted p53-response elements in promoter regions of (A) a known p53 transcriptional target, Cdkn1a , and (B) the novel target, Ins2 . Percent input at each predicted response element is calculated from the standard input curve. (TIF) [file pone.0028808.s007.tif]

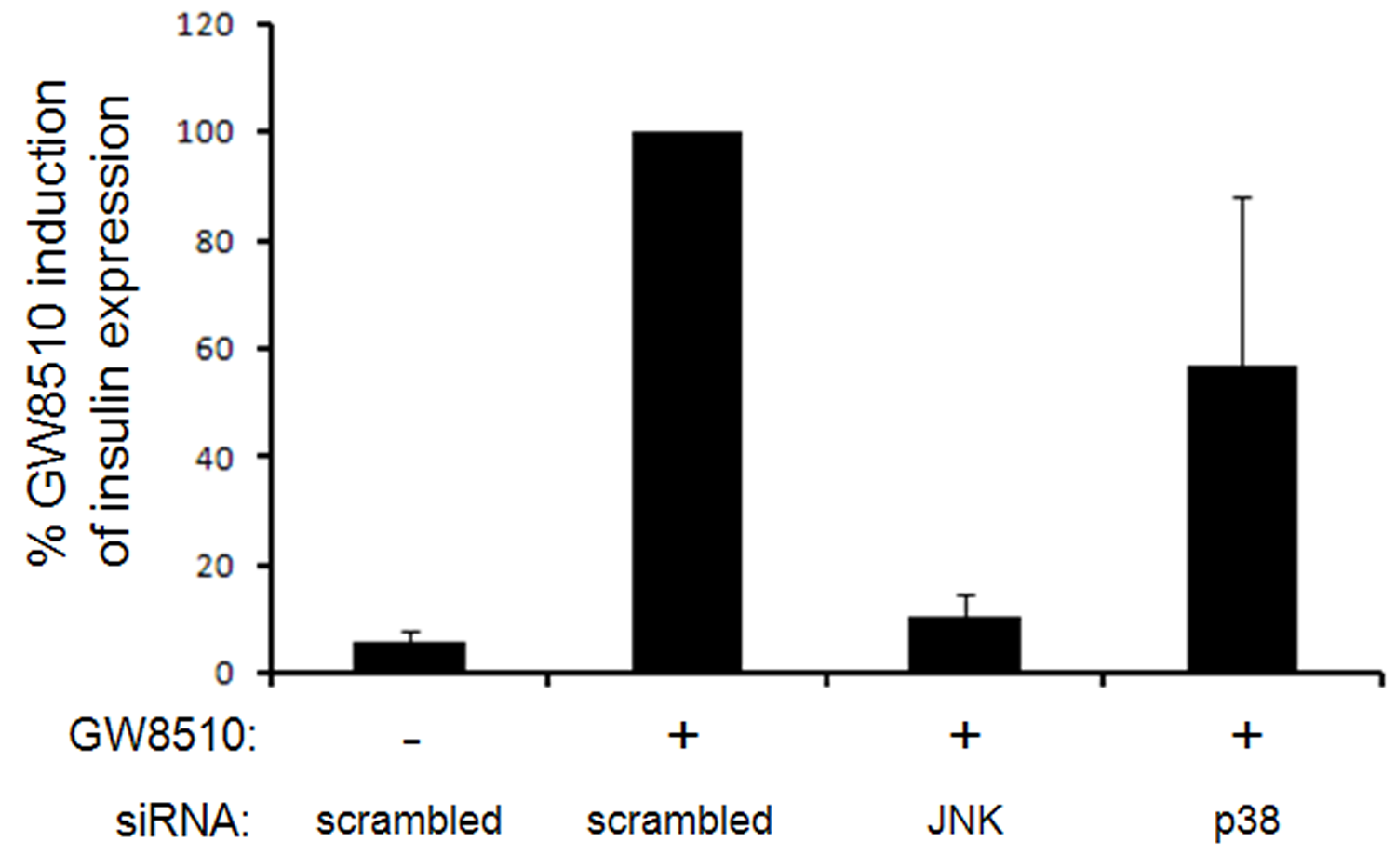

Supplement: Figure S8 — Knock-down of JNK and p38 inhibit induction of Ins2 by GW8510. Alpha cells were transfected with the indicated siRNAs for one day, followed by three-day treatment with 1.65 µM GW8510. mRNA was collected for analysis of Ins2 gene expression by quantitative PCR, using actin as a normalization control. (TIF) [file pone.0028808.s008.tif]

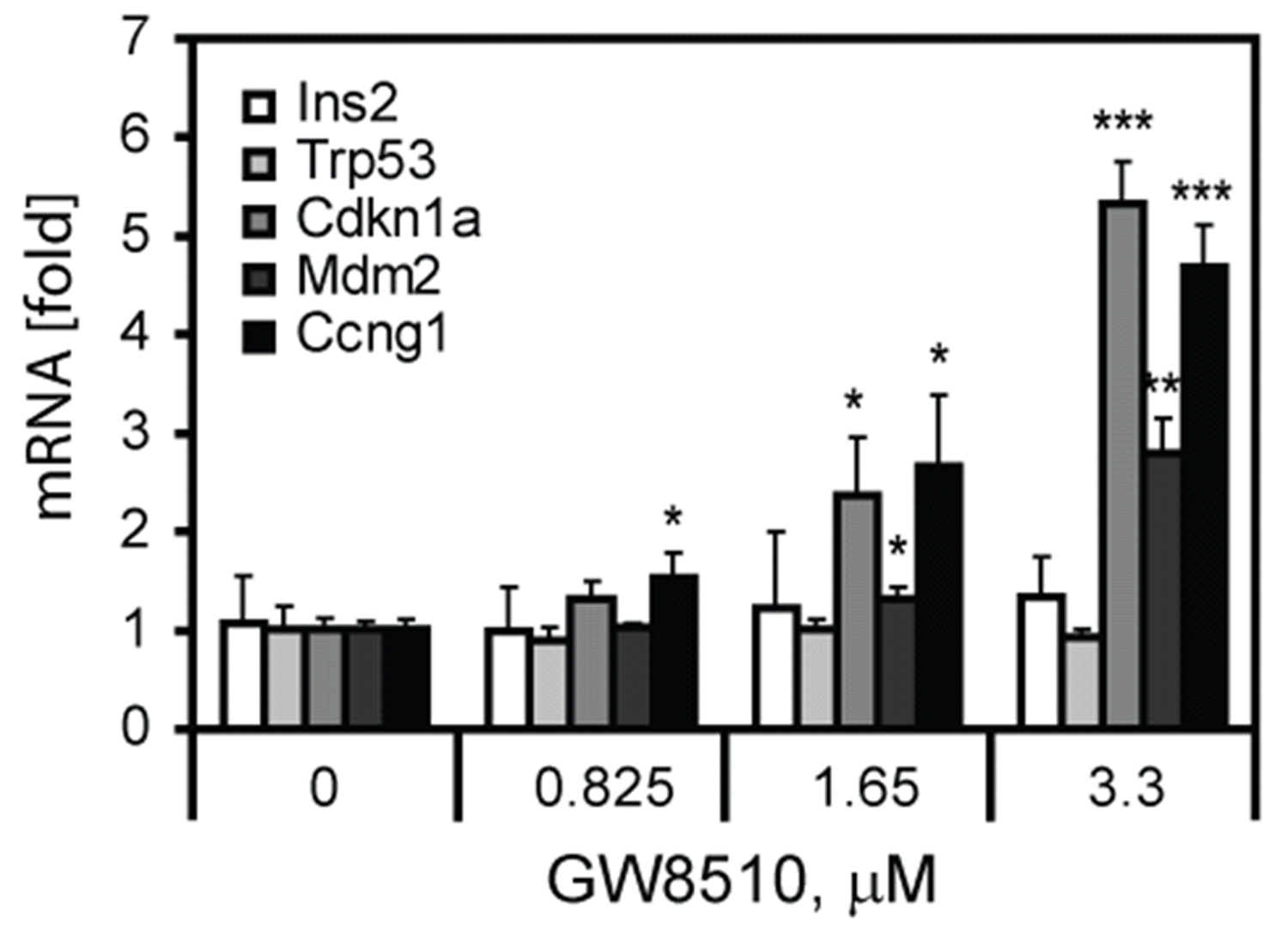

Supplement: Figure S9 — Effects of GW8510 on NIH3T3 cells. Gene expression changes in NIH3T3 mouse embryonic fibroblasts following a 3-day treatment of aTC1 cells GW8510 at indicated concentrations. Data represent the mean±SD of three biological replicates; *p<0.05, **p<0.01 and ***p<0.001. (TIF) [file pone.0028808.s009.tif]
