# Supplementary material for: GW8510 Increases Insulin Expression in Pancreatic Alpha Cells through Activation of p53 Transcriptional Activity
Source: PLoS One. 2012 Jan 5;7(1):e28808. doi: 10.1371/journal.pone.0028808 (PMC3252286; doi:10.1371/journal.pone.0028808)
Supplement: Table S1 — p53-pathway related GSEA gene sets found to be enriched following 5-day treatment of alpha cells with GW8510. (DOC) [file pone.0028808.s010.doc]

**Table S1. p53-pathway related GSEA gene sets found to be enriched following 5-day treatment of alpha cells with GW8510**

| **GeneSet NAME** | **GeneSet SIZE** | **ES** | **NES** | **FDR q-val** |
| --- | --- | --- | --- | --- |
| MACLACHLAN_BRCA1_TARGETS_UP | 15 | 0.499 | 1.908 | 0.054 |
| TANG_SENESCENCE_TP53_TARGETS_UP | 19 | 0.682 | 1.704 | 0.077 |
| INGA_TP53_TARGETS | 15 | 0.661 | 1.645 | 0.077 |
| P53PATHWAY | 16 | 0.631 | 1.692 | 0.082 |
| P53GENES_ALL | 15 | 0.661 | 1.646 | 0.085 |
| BRCA1_OVEREXP_UP | 140 | 0.373 | 1.659 | 0.090 |
| HSA04115_P53_SIGNALING_PATHWAY | 61 | 0.605 | 1.582 | 0.107 |
| P53HYPOXIAPATHWAY | 18 | 0.574 | 1.512 | 0.113 |
| P53_SIGNALING | 81 | 0.379 | 1.458 | 0.119 |
| P53_BRCA1_UP | 32 | 0.840 | 1.313 | 0.164 |
