# Supplementary material for: GW8510 Increases Insulin Expression in Pancreatic Alpha Cells through Activation of p53 Transcriptional Activity
Source: PLoS One. 2012 Jan 5;7(1):e28808. doi: 10.1371/journal.pone.0028808 (PMC3252286; doi:10.1371/journal.pone.0028808)
Supplement: Table S2 — CisRED prediction of p53 response elements (group 200034) in promoter regions of selected genes from the Mouse 4.0 database. (DOC) [file pone.0028808.s011.doc]

**Table S2. CisRED prediction of p53 response elements (group 200034) in promoter regions of selected genes from the Mouse 4.0 database**

| **Index** | **Atomic motif ID (craMmus)** | **Annotation p-value** | **Discovery p-value** | **Motif location** | **Strand** | **Name(s) associated with the search region that contains this motif** |
| --- | --- | --- | --- | --- | --- | --- |
| 1 | 73690 | 6.93E-07 | 0.00424 | **chr11: 40,568,967-40,568,978** | + | ENSMUSG00000020326 **Ccng1** |
| 90 | 126631 | 4.96E-05 | 0.00279 | **chr4: 11,083,410-11,083,421** | - | ENSMUSG00000028211 **Trp53inp1** |
| 188 | 94617 | 1.01E-04 | 0.0632 | **chr17: 29,228,764-29,228,789** | + | ENSMUSG00000023067 **Cdkn1a** |
| 227 | 288 | 1.22E-04 | 0.0217 | **chr7: 149,866,899-149,866,909** | + | ENSMUSG00000000215 **Ins2** |
| 307 | 126539 | 1.60E-04 | 0.0231 | **chr4: 11,115,902-11,115,909** | - | ENSMUSG00000028212 **Ccne2** |
| 380 | 197551 | 1.91E-04 | 0.000973 | **chr4: 88,940,985-88,940,990** | + | ENSMUSG00000044303 **Cdkn2a** |
