# Supplementary material for: GW8510 Increases Insulin Expression in Pancreatic Alpha Cells through Activation of p53 Transcriptional Activity
Source: PLoS One. 2012 Jan 5;7(1):e28808. doi: 10.1371/journal.pone.0028808 (PMC3252286; doi:10.1371/journal.pone.0028808)
Supplement: Table S3 — Quantitative real-time PCR primers for indicated mouse genes. (DOC) [file pone.0028808.s012.doc]

**Table S3. Quantitative real-time PCR primers for indicated mouse genes**

| **Gene** | **Fw primer sequence** | **Rv primer sequence** |
| --- | --- | --- |
| Ins2 | GTCAAGCAGCACCTTTGTGGTTCC | ACAATGCCACGCTTCTGCTG |
| Pdx1 | TCCACCACCACCTTCCAGCTCA | AATTCCTTCTCCAGCTCCAG |
| Gcg | TTCCTTTGCTGCCTGGCCCT | TTCCCAGACAGAAGCGCATGAG |
| Pax6 | CACCATGCAGAACAGTCACAGCGGAGTGAATC | TTACTGTAATCGAGGCCAGTACTGAGA |
| p53 | CGCGGGCGTAAACGCTTCGA | TCTTCAGGTAGCTGGAGTGAGCCC |
| Ccng1 | GCGTTGGAGATCCAAGCACTGA | GGAAACAAGCTCTTGCCAGAAGG |
| Cdkn1a | TCGCTGTCTTGCACTCTGGTGT | CCAATCTGCGCTTGGAGTGATAG |
| Mdm2 | CCGAGTTTCTCTGTGAAGGAGC | GTCTGCTCTCACTCAGCGATGT |
| Ccnd1 | GCAGAAGGAGATTGTGCCATCC | AGGAAGCGGTCCAGGTAGTTCA |
| Ccnd2 | GCAGAAGGACATCCAACCGTAC | ACTCCAGCCAAGAAACGGTCCA |
| Bid | CCACAACATTGCCAGACATCTCG | TCACCTCATCAAGGGCTTTGGC |
| Apaf1 | CACGAGTTCGTGGCATATAGGC | GGAAATGGCTGTCGTCCAAGGA |
| Gtse1 | AGAGGATCACCAGCAAGCTCCA | GTTTCGTCCTCTGAATGCTGGC |
| Zmat3 | AGAACCATGCCAAGAGACTCCG | TCACTCCCTTCTTTCCGAGTCC |
| Cdkn2a | TGTTGAGGCTAGAGAGGATCTTG | CGAATCTGCACCGTAGTTGAGC |
| Cdkn2b | ATCCCAACGCCCTGAACCGCT | AGTTGGGTTCTGCTCCGTGGAG |
| Ei24 | CTTGGCTTTCCTCACAGCAATGC | GGAGTCTTTGCTTCATTGGCGC |
| Ctsd | TAAGACCACGGAGCCAGTGTCA | CCACAGGTTAGAGGAGCCAGTA |
| Atr | GAAAGAGGCTCCTACCAACGAG | CAACTGTCACCTGGAGACTTGC |
| Gadd45a | CCTGGAGGAAGTGCTCAGCAAG | GTCGTCTTCGTCAGCAGCCAG |
| Cdkn1b | AGCAGTGTCCAGGGATGAGGAA | TTCTTGGGCGTCTGCTCCACAG |
| Igf1 | GTGGATGCTCTTCAGTTCGTGTG | TCCAGTCTCCTCAGATCACAGC |
| Thbs1 | GGTAGCTGGAAATGTGGTGCGT | GCACCGATGTTCTCCGTTGTGA |
| Atf3 | GAAGATGAGAGGAAAAGGAGGCG | GCTCAGCATTCACACTCTCCAG |
| Actb | GGTGGGAATGGGTCAGAAGGAC | GGCCACACGCAGCTCATTGT |
| Gapdh | TGTGCAGTGCCAGCCTCGTC | TGCCACTGCAAATGGCAGCC |
