# Supplementary material for: GW8510 Increases Insulin Expression in Pancreatic Alpha Cells through Activation of p53 Transcriptional Activity
Source: PLoS One. 2012 Jan 5;7(1):e28808. doi: 10.1371/journal.pone.0028808 (PMC3252286; doi:10.1371/journal.pone.0028808)
Supplement: Table S4 — Primers used for PCR analysis of putative p53 response elements predicted by CisRED. (DOC) [file pone.0028808.s013.doc]

**Table S4.** **Primers used for PCR analysis of putative p53 response elements predicted by CisRED**

| **Promoter of gene** | **Fw primer sequence** | **Rv primer sequence** | **Fragment size** |
| --- | --- | --- | --- |
| Ins2 | TGTCATTCCTCATGGAGACG | TGGCTCTCCTTGGGAACTTA | 188bp |
| Cdkn1a | GAAGGGGGTCCTTCAACTGT | TCCTGCTTTGGAGAAGCTGT | 208bp |
| Ccng1 | TACATTCTGGTCCGCTACCC | CCCGTTGTCCAGACTTCAAT | 184bp |
