# Supplementary material for: GW8510 Increases Insulin Expression in Pancreatic Alpha Cells through Activation of p53 Transcriptional Activity
Source: PLoS One. 2012 Jan 5;7(1):e28808. doi: 10.1371/journal.pone.0028808 (PMC3252286; doi:10.1371/journal.pone.0028808)
Supplement: Table S5 — Species comparisons of orthologous sequences of the p53 response element predicted in mouse Ins2 promoter region by CisRED. (DOC) [file pone.0028808.s014.doc]

**Table S5. Species comparisons of orthologous sequences of the p53 response element predicted in mouse Ins2 promoter region by CisRED**

| **Ensembl gene ID** | **Type** | **Species** | **Position** | **Std** | **(+)Sequence** | **Std** | **(-)Sequence** |
| --- | --- | --- | --- | --- | --- | --- | --- |
| ENSMUSG00000000215 | Target | Mus musculus | **chr7:149,866,899-149,866,909** | + | ACCCTGAGCAT | – | **ATGCTCAGGGT** |
| ENSMUSG00000000215_RNOR | Orthologue | Rattus norvegicus | **chr1:203,125,890-203,125,900** | + | ACCCTGAGCAT | – | **ATGCTCAGGGT** |
| ENSMUSG00000000215_PTRO | Orthologue | Pan troglodytes | chr9:2,335,443-2,335,453 | + | ACCCTGAGCCC | – | GGGCTCAGGGT |
| ENSMUSG00000000215_HSAP | Orthologue | Homo sapiens | **chr11:2,138,935-2,138,945** | + | ATCCTGAGCCC | – | **GGGCTCAGGAT** |
| ENSMUSG00000000215_MMUL | Orthologue | Macaca mulatta | SCAFFOLD150906:857-867 | – | GCCCTGAGCCC | + | GGGCTCAGGGC |
| ENSMUSG00000000215_BTAU | Orthologue | Bos taurus | chr29:44,350,558-44,350,568 | + | ACCCTGAGCCC | – | GGGCTCAGGGT |
| ENSMUSG00000000215_CFAM | Orthologue | Canis familiaris | chr18:49,347,846-49,347,856 | + | ACCCTGAGCCC | – | GGGCTCAGGGT |
| ENSMUSG00000000215_OCUN | Orthologue | Oryctolagus cuniculus | scaffold_96961:504-514 | – | GGGCTGAGCCT | + | AGGCTCAGCCC |
| ENSMUSG00000000215_CPOR | Orthologue | Cavia porcellus | ENSMUSG00000000215_CPOR:207-217 | – | GGCGTCAGCAC | + | GTGCTGACGCC |
